# ICAM-1-mediated Src signaling pathway plays a pivotal role in encephalomyocarditis virus entry

**DOI:** 10.1128/jvi.00715-25

**Published:** 2025-07-09

**Authors:** Ruiya Lian, Xueer Dou, Na Wang, Shasha Li, Jingying Xie, Xiangrong Li, Yanmei Yang, Yanqiao Wen, Huixia Li, Ruofei Feng

**Affiliations:** 1 Engineering Research Center of Key Technology and Industrialization of Cell-based Vaccine, Ministry of Education, Biomedical Research Center, Northwest Minzu University 66293, Lanzhou, China; 2 Key Laboratory of Biotechnology and Bioengineering of State Ethnic Affairs Commission, Biomedical Research Center, Northwest Minzu University 66293, Lanzhou, China; 3 Gansu Tech Innovation Center of Animal Cell, Biomedical Research Center, Northwest Minzu University 66293, Lanzhou, China; 4 School of Life Sciences and Engineering, Northwest Minzu University 66293, Lanzhou, China; Loyola University Chicago - Health Sciences Campus, Maywood, Illinois, USA

**Keywords:** EMCV, virus entry, Src, Ezrin, Cav-1, ICAM-1

## Abstract

Encephalomyocarditis virus (EMCV), a potential zoonotic pathogen with a broad host range, has been reported to enter cells via caveolin-mediated endocytosis (CavME), a process initiated by caveolae detachment from the plasma membrane. However, the molecular mechanism underlying EMCV’s cellular entry remains incompletely characterized. Here, we identified for the first time that the overexpression of Src kinase and Fyn kinase individually enhanced the replication and proliferation of EMCV. The treatment of specific small interfering RNA or inhibitors resulted in the downregulation of EMCV internalization, indicating the involvement of Src, Fyn, and Ezrin in the viral entry process. By treating different cell lines (BHK-21, hCMEC/D3, and PK15 cells) from various species with inhibitors of Src or Ezrin, as well as using siCav-1, and subsequently performing EMCV invasion experiments, we demonstrated the sequential activation of Src, Ezrin, and Cav-1 during EMCV internalization, indicating that this signaling pathway is widely present during EMCV infection of human-, hamster-, and pig-derived cells. Interestingly, the candidate receptor protein ICAM-1 interacts with Src and regulates its phosphorylation. Compared to the wild-type cells, lower levels of the phosphorylation of Src, Ezrin, and Cav-1 induced by EMCV were observed in ICAM-1 knockout BHK-21 cells. These findings indicate that EMCV entry into BHK-21 cells relies on the activation of the ICAM-1-Src-Ezrin-Cav-1 pathway. Our study first elucidates the molecular mechanism of EMCV invading into BHK-21 cells, providing potential targets for limiting EMCV infection and further refining the regulatory mechanism of the Src pathway on CavME.

**IMPORTANCE:**

EMCV has a wide host range and is a potential zoonotic pathogen, yet the mechanism of its entry into cells remains unclear. This study has found that the host proteins Src, Fyn, and Ezrin are involved in viral replication and proliferation, and their activity inhibitors hinder EMCV invasion into cells. With the participation of the cell surface adhesion factor ICAM-1, EMCV infects host cells by sequentially activating the phosphorylation of Src, Ezrin, and Cav-1, mediating the entry of EMCV into host cells through CavME. We have demonstrated for the first time the existence of an ICAM-1-Src-Ezrin-Cav-1 signaling pathway during EMCV entry into susceptible cells, which mediates the internalization process of the virus. Our research not only unravels the molecular mechanism of EMCV invasion, filling a gap in related research, but also provides a theoretical basis for the development of specific antiviral therapeutics.

## INTRODUCTION

As a typical small RNA virus, encephalomyocarditis virus (EMCV) has a single, positive-stranded RNA genome encapsulated by capsid proteins and lacks an envelope structure (1). This pathogen can infect a wide range of vertebrate animals, including humans (2), wild animals (3, 4) and domesticated animals (5, 6), making it a potential zoonotic pathogen. Clinically, EMCV primarily causes diseases such as encephalitis, myocarditis, and diabetes, posing a significant threat to both human and veterinary public health (1, 2). Furthermore, EMCV is widely used as a natural immune stimulant in laboratories worldwide, which further increases the likelihood of cross-species infection and enhances the virulence of this pathogen. Therefore, elucidating the life cycle of EMCV is crucial for discovering new antiviral drug targets.

Despite the simplicity of their structure and composition, viruses exhibit complex life-cycle processes within host cells. Each stage of viral infection entails numerous interactions between viral and host proteins, accompanied by a series of intricate cellular processes (7). The BHK-21 cell line is frequently utilized for the isolation and propagation of EMCV, as well as for its related research on infection mechanisms (8–10). In our laboratory, a previous study discovered that the entry of EMCV into BHK-21 cells does not rely on clathrin-mediated endocytosis (CME) or macropinocytosis. Instead, it depends on caveolin-mediated internalization (10), a mechanism that resembles the entry pathway of foot-and-mouth virus as previously documented in research (11). However, there was no further exploration into the molecular mechanism of EMCV entry into target cells. Another picornavirus, group B coxsackieviruses (CBVs), has been found to interact with the glycosylphosphatidylinositol-anchored protein decay-accelerating factor, which activates two non-receptor tyrosine kinases: Abl kinase and Fyn kinase. The activation of these two kinases subsequently leads to the phosphorylation of caveolin, facilitating the transport of the virus into the cell within caveolar vesicles (12). Similarly, the infection of avian reovirus (ARV) (13), Newcastle disease virus (NDV) (14), and Japanese encephalitis virus (JEV) (15) can also activate Src kinase, which in turn promotes virus internalization. Notably, researchers have discovered that three proteins—Src, Ezrin (a member of the ERM protein family), and Caveolin-1 (Cav-1)—undergo sequential phosphorylation to form a complex that mediates the internalization of JEV (15).

This study aims to investigate whether non-receptor tyrosine kinases, such as Fyn and Src, are activated during the entry of EMCV into cells and to explore whether they exhibit any cascade relationships in the process of viral internalization. Initially, we identified the relationships between the three proteins (Fyn, Src, and Ezrin) and EMCV infection, as well as EMCV’s entry into cells. Additionally, we explored the cascade relationship between these proteins and ICAM-1, a potential receptor protein for EMCV that was identified in our laboratory (Z. Y. Hou et al., unpublished data). This study represents the first systematic exploration of the mechanism underlying EMCV’s entry into BHK-21 cells, setting the stage for a deeper understanding of EMCV’s entry and offering multiple novel targets for antiviral drug development.

## MATERIALS AND METHODS

### Cell lines

Human embryonic kidney cells (HEK 293T) were maintained in Dulbecco’s modified Eagle medium (DMEM), supplemented with 10% fetal bovine serum (FBS) sourced from Minhai Bio (Lanzhou, China), under conditions of 37°C and 5% CO_2_. Pig kidney cell line (PK15), baby hamster kidney cells (BHK-21), and BHK-21 cells with ICAM-1 knockout (KO) were cultivated using 10% new calf serum (NBS), also obtained from Minhai Bio. In contrast, human lung adenocarcinoma alveolar basal epithelial cells (A549) were preserved in F12 medium, enriched with 15% NBS. All of these cell lines are currently stored and maintained by our laboratory. Immortalized human cerebral microvascular endothelial cell line (hCMEC/D3) was purchased from BNCC Bio (Henan, China) and maintained in endothelial cell medium supplemented with 5% FBS, 1% endothelial cell growth supplement, and 1% penicillin-streptomycin.

### Plasmids, small interfering RNAs, antibodies, and reagents

Recombinant plasmids encoding human genes, including Myc-Src, Myc-Fyn, and HA-Cav-1, were designed and then synthesized by GenScript (Nanjing, China). A Flag-tagged ICAM-1 (from *Mus musculus*) expression plasmid was constructed and is currently stored within our laboratory. Additionally, the empty vectors (EVs) pcDNA3.1-Myc-His, pCMV-3Tag, and pCMV-HA serve as negative control plasmids and were provided by our laboratory. The small interfering RNA (siRNA) sequences targeting the human *Fyn* and *Src* genes [siFyn-1236: 5′-GGUGGAUACUACAUUACCA(dTdT)-3′ and siSrc-888: 5′-CAGGCUGAGGAGUGGUAUU(dTdT)-3′] were referenced from prior research (16, 17), whereas the siRNA sequences targeting the Cav-1 gene [siCav-1: 5′-GCAACATCTACAAGCCCAA(dTdT)-3′] in BHK21 cells were derived from an earlier research in our laboratory (10). Three specific siRNAs and a negative control siRNA (siNC) were synthesized by Accurate Biology.

A mouse monoclonal antibody (Mab) targeting the EMCV VP1 protein was prepared by GeneCreate (Wuhan, China). Additionally, a Mab targeting β-tubulin (TA503129) was acquired from OriGene Technologies (Maryland, USA). Polyclonal antibodies against Flag-tag (20543-1-AP), Myc-tag (60003-2-Ig/10828-1-AP), HA-tag (51064-2-AP), Caveolin-1 (16447-1-AP), Src (11097-1-AP), and ICAM-1 (60299-1-Ig) were purchased from Proteintech Biotechnology (Wuhan, China). Antibodies against Mouse IgG1 (5415S), HA-tag (3724), phospho-Caveolin-1 (Tyr14) (3251S), and Phospho-Ezrin (3726S) were obtained from Cell Signaling Technology (Cambridge, UK). Antibodies against Src (sc-8056), phospho-Src (sc-81521) targeting Tyr419 (human) or Tyr424 (mouse), Ezrin (sc-271541), Fyn (sc-434), phospho-Fyn (sc-377555), Caveolin-1 (sc-53564), phospho-Caveolin-1 (sc-373836), and Rab5 (sc-46692) were acquired from Santa Cruz Biotechnology (Dallas, USA). Antibodies against Rab7 (R25524), Rab9 (R25527), and Rab11 (R380914) were sourced from ZEN-Bioscience (Chengdu, China). ICAM-1 Polyclonal Antibody (PA5-96365) was purchased from Invitrogen (California, USA). Peroxidase- and Cy3-labeled goat antimouse IgG (H + L) antibodies, as well as 488-labeled goat antirabbit IgG (H + L) antibodies, were acquired from Jackson ImmunoResearch Inc. (Pennsylvania, USA).

The reagents acquired from Selleck (Texas, USA) include PP2 (S7008), a potent inhibitor of Src family kinases with specificity for Lck/Fyn, and two inhibitors of Src activation, namely, Dasatinib (S1021) and Saracatinib (S1006), both of which exhibit inhibitory activity against Y419 phosphorylation (18, 19). Additionally, NSC668394 (382605-72-3), an effective inhibitor of Ezrin (Thr567) phosphorylation, was sourced from Merck (Darmstadt, Germany) and utilized in this study. Methyl-β-cyclodextrin (MβCD), a cholesterol inhibitor, was acquired from Sigma-Aldrich (Missouri, USA). The Cell Proliferation and Cytotoxicity Assay Kit (CCK-8) (Enhanced, MA0218) was purchased from Meilunbio (Dalian, China) to assess the cytotoxicity of the aforementioned inhibitors.

### Virus propagation and titration

The EMCV strain (PV21), preserved within our laboratory, was propagated in BHK-21 cells. Upon the emergence of cytopathic effects (CPEs), the cell culture was harvested, subjected to three cycles of freeze-thaw, and then centrifuged to eliminate cell debris. The resultant supernatant, containing the virus stock, was stored at −80°C. The virus titer was determined using BHK-21 cells. Initially, serial 10-fold dilutions of the EMCV stock were added (100 µL/well) to BHK-21 cells that were 90% confluent in a 96-well plate. After a 1 h incubation, the culture medium was aspirated, and DMEM supplemented with 3% NBS was added. The number of wells exhibiting CPE was counted at 3–5 days post-infection, and the virus titer was calculated employing Karber’s method.

### Infection, adsorption, and entry of EMCV

In EMCV infection experiments, considering that EMCV-induced CPE compromises the collection of cellular samples, different multiplicities of infection (MOIs) were inoculated, depending on experimental objectives. For instance, a lower MOI was employed to monitor viral proliferation kinetics over extended time periods, whereas a higher MOI was utilized to detect viral antigens during early infection stages (e.g., adsorption and internalization phases). A549 cells subjected to different treatments were infected at an MOI of 0.00001. Subsequent to a 1 h incubation, the culture medium was aspirated and replenished with DMEM supplemented with 3% NBS. Cells were harvested at 12, 24, and 36 h post-infection (hpi) to evaluate viral replication and propagation. To elucidate the replication and proliferation dynamics of EMCV under various treatment conditions, BHK-21 cells were infected with EMCV at an MOI of 0.1. At designated post-infection time points (e.g., 6, 9, and 12 h), cellular lysates were harvested for Western blot analysis to detect the viral protein VP1. Besides, to evaluate the prolonged effects of NSC668394 treatment on EMCV propagation and virus-induced CPE, BHK-21 cells were infected with a low MOI (0.000001) of EMCV. Microscopic examination of CPE was performed at 12, 24, 36, and 48 hpi, followed by cell collection for quantitative analysis of viral genomic copies. Additionally, parallel cultures from replicate wells were sampled to quantify viral genome copies and viral titers.

In the virus adsorption assay, BHK-21 cells were pre-cooled at 4°C for 15 min prior to the inoculation of EMCV (MOI of 3). The cells were then incubated with the virus at 4°C for 1 h, a temperature that arrests endocytosis, thereby allowing for the assessment of virus binding alone. Unbound virus particles were meticulously removed through three washes with pre-chilled phosphate-buffered saline (PBS). Subsequently, the cells were collected, and total RNA was extracted to determine the relative abundance of the EMCV 3D gene using the reverse transcription quantitative PCR (RT-qPCR) method.

The internalization assay was adapted from previously established protocols (20, 21). Briefly, BHK-21 cells were inoculated with EMCV and incubated at 4°C for 1 h to allow virus adsorption. Unbound viral particles were removed by three washes with ice-cold PBS. Pre-warmed DMEM supplemented with 3% NBS was then added to the wells, and the cells were shifted to a 37°C incubator for 15, 30, or 60 min to facilitate viral internalization. To minimize residual non-internalized virions on the cell surface (note: this treatment may not fully eliminate all surface-bound particles), the cells were treated with 0.25% trypsin on ice for 3 min, followed by three additional washes with ice-cold PBS. Ultimately, the cells were harvested and assayed for the relative level of viral genes using RT-qPCR. The experimental protocols for EMCV internalization assays in A549 and PK15 cells were consistent with those established for BHK-21 cells. However, based on our laboratory’s prior findings demonstrating that hCMEC/D3 cells exhibit significantly lower susceptibility to EMCV infection compared to BHK-21 cells, viral internalization assays in hCMEC/D3 cells were performed using an elevated MOI of 10 to compensate for the reduced infectivity.

### RT-qPCR and TaqMan probe RT-qPCR

Total RNA was extracted from the cells utilizing the TransZol kit (ET101-01-V2; TransGen Biotech, Beijing, China) and subsequently reverse transcribed into cDNA with the PrimeScript RT kit (RRO47A; TaKaRa Bio, Kusatsu, Shiga, Japan). RT-qPCR was conducted using TB Green Premix Ex Taq II (Tli RNaseH Plus) (RR820A; TaKaRa Bio, Beijing, China), with the results analyzed using the 2-ΔΔCT method. GAPDH mRNA served as the internal control for normalization. The primer sequences employed for amplifying the target genes in RT-qPCR were as follows: MoGAPDH-qF (forward primer): 5′-AAGGCCATCACCATCTTCCA-3′, MoGAPDH-qR (reverse primer): 5′-GCCAGTAGACTCCACAACATAC-3′; EMCV-3D-qF (forward primer for EMCV 3D gene): 5′-GTCATACTATCGTCCAGGGACTCTAT-3′, EMCV-3D-qR (reverse primer for EMCV 3D gene): 5′-CATCTGTACTCCACACTCTCGAATG-3′. Additionally, TaqMan probe-based RT-qPCR, as described in references 22 and 23, was utilized to quantify viral copies in infected cells. This method involved the use of a specific EMCV probe (5′-CACTTCGATCACTATGCTTGCCGTT-3′), along with the aforementioned EMCV-3D-qF and EMCV-3D-qR primers.

### Co-immunoprecipitation assay

Cell lysates were prepared using High Efficiency RIPA Lysis Buffer (R0010; Solarbio, Beijing, China), supplemented with a Protease and Phosphatase Inhibitor Cocktail (P1045, Beyotime, Shanghai, China). For immunoprecipitation, 2–4 μg of antibody was incubated with 20–25 μL of Protein G Agarose at 4°C for a duration of 4–6 h. Following this incubation, the cell lysate was added and incubated overnight. The Protein G Agarose beads were then washed five times with pre-cooled PBS solution (adjusted to pH 7). The final immunoprecipitated complex was analyzed using Western blotting.

### Indirect immunofluorescence assays

The BHK21 cells not treated or treated with different inhibitors on cell slides were infected with EMCV at an MOI of 3. At different time points after infection, the cells were fixed with 4% paraformaldehyde and permeabilized with 0.25% Triton X-100, followed by blocking with 5% bovine serum albumin in PBS. Additionally, the blocked cells were incubated with specific antibodies for target proteins in a 37°C incubator for 1 h. After washing five times with PBS, the cells were co-incubated with a mixture of two labeled antibodies, Cy3-conjugated goat antimouse IgG (H + L) and 488-conjugated goat antirabbit IgG (H + L), at 37°C for 1 h. Subsequently, the cell nuclei were stained with 4′,6-diamidino-2-phenylindole (DAPI). All the steps were performed at room temperature. Finally, the cells were observed, and a single Z-plane was captured under a confocal microscope (LSM 900 with Airyscan 2).

### Statistical analysis

The data obtained from both RT-qPCR and TaqMan probe RT-qPCR assays were analyzed utilizing the GraphPad Prism 9.0 software package (GraphPad Software, San Diego, CA, USA). Statistical evaluations were performed using either Student’s *t*-tests or one-way analysis of variance, depending on the nature of the comparisons being made. The results were presented as the mean ± standard deviation, reflecting data aggregated from at least three independent experiments to ensure robustness and reproducibility. The statistical significance levels in the figures are indicated by asterisks (*** for *P* < 0.001, ** for *P* < 0.01, and * for *P* < 0.05), with *P* < 0.05 serving as the threshold for statistical significance.

## RESULTS

### Fyn kinase is involved in the replication and invasion of EMCV

**Fig 1:**
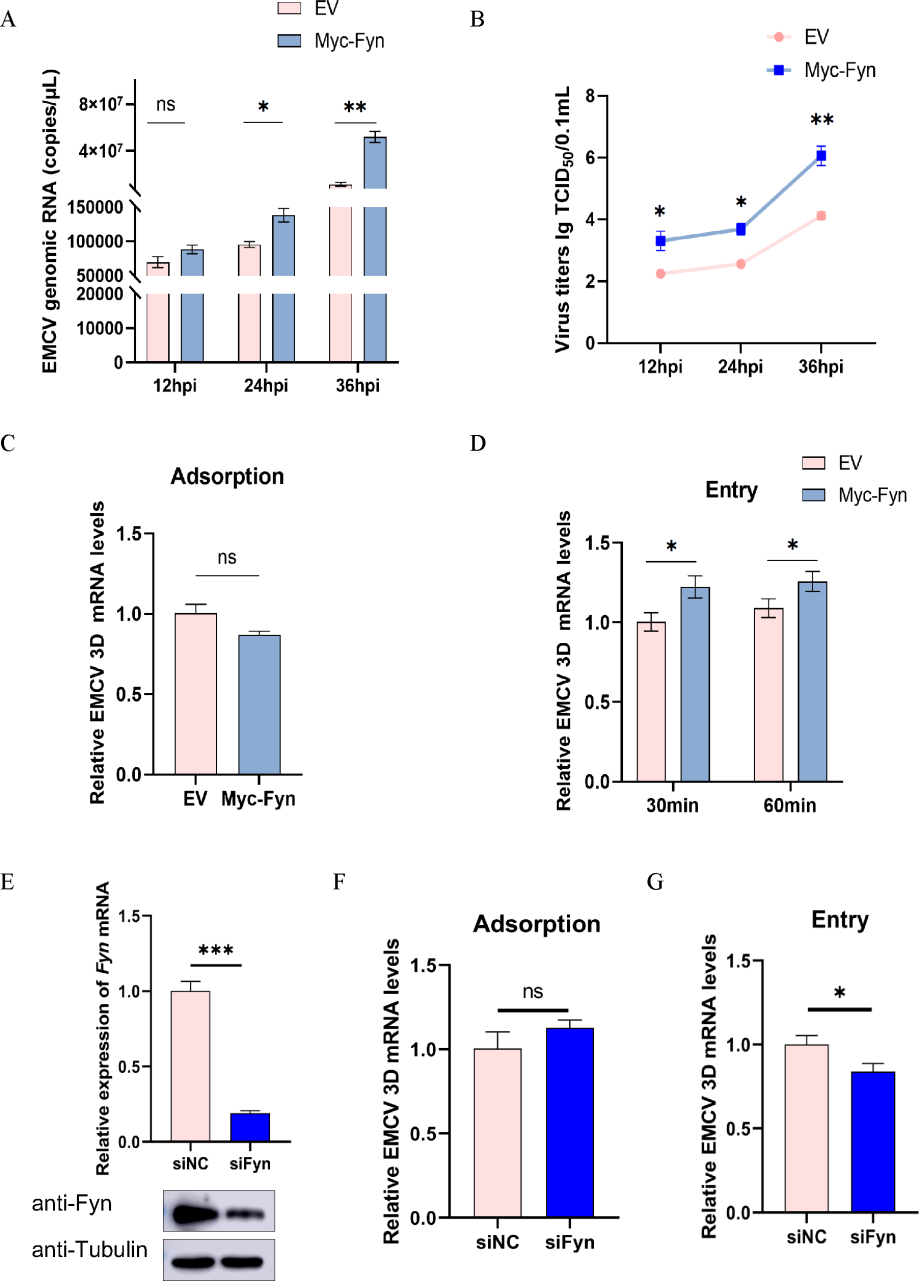
Overexpression of Fyn promotes the replication and entry of EMCV. (**A**) A549 cells were transfected with either the empty vector (EV) or Myc-Fyn. Following a 24 h incubation period, EMCV (at an MOI of 0.00001) was inoculated. Cells were harvested at 12, 24, and 36 h post-infection for the extraction of viral RNA. The mRNA levels of the EMCV 3D gene were detected using TaqMan probe-based RT-qPCR. (**B**) Virus titers were also determined using the TCID_50_ method. (**C** and **D**) A549 cells were transfected with a Myc-tagged Fyn plasmid, with an EV control group included for comparison. Twenty-four hours later, EMCV (at an MOI of 3) was used to conduct the adsorption and internalization experiments. The treated cells were collected, and total RNA was extracted. Subsequently, the relative levels of EMCV 3D mRNA were measured by RT-qPCR. All genes detected in the RT-qPCR were normalized to GAPDH expression. (**E**) Two small interfering RNAs, siFyn-1236 specifically targeting human *Fyn* gene and negative control siRNA (siNC), were respectively transfected into A549 cells. After 24 h, the cells were harvested, and Fyn expression was independently detected using Western blotting (bottom panel) and RT-qPCR (top panel). The adsorption (**F**) and internalization (**G**) experiments were performed in siRNA-transefected A549 cells. Cells were subjected to viral mRNA detection 30 min following viral internalization. Statistical significance was indicated as follows: ***P < 0.001, **P < 0.01, *P < 0.05. ns, no significant difference.

Building upon previous discoveries that implicate the Fyn kinase in the entry of picornaviruses, particularly CBVs (12), our objective is to investigate whether Fyn kinase is similarly involved in the infection or cellular entry of EMCV. Initially, we designed and synthesized a plasmid expressing the recombinant Myc-tagged Fyn protein. After sequentially transfecting with the plasmid and subsequently infected with EMCV in A549 cells, viral copies and titers were assayed at three time points post-infection: 12, 24, and 36 h. As illustrated in Fig. 1A, compared to the EV control group, there is a notable increase in viral copies within the cells transfected with Myc-Fyn at 24 and 36 hpi. Consistent with this observation, the viral titers were also significantly elevated in Myc-Fyn-expressing cells at 12, 24, and 36 hpi, respectively (Fig. 1B).

To investigate whether Fyn kinase influences the entry of EMCV into target cells, virus adsorption and internalization experiments were conducted in A549 cells transfected with EV or Myc-Fyn. The relative mRNA levels of the EMCV 3D gene were assessed to evaluate viral adsorption and internalization. The findings revealed that, in comparison to the EV control, there was no significant change in the relative mRNA level of the EMCV 3D gene in the Myc-Fyn group during viral adsorption (Fig. 1C). However, the results of the internalization experiments demonstrated that the overexpression of Fyn significantly enhanced the entry of EMCV into A549 cells (Fig. 1D). To clarify Fyn’s role in EMCV invasion, we efficiently knocked down Fyn expression using its specific siRNA, reducing its expression at both mRNA and protein levels (Fig. 1E). This depletion notably suppressed viral internalization but not adsorption (Fig. 1F and G).

### The inhibition of Fyn kinase decreases EMCV replication and internalization

**Fig 2:**
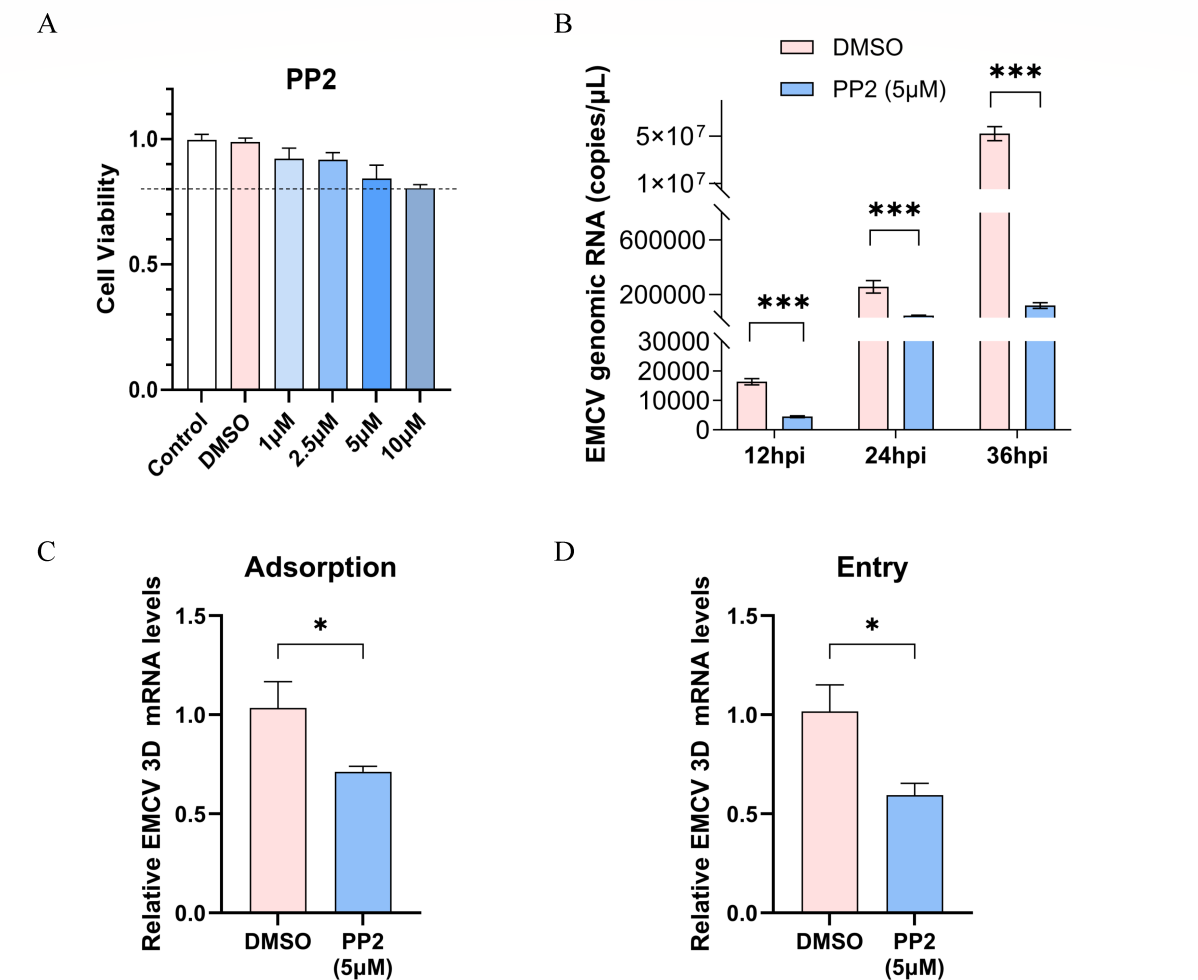
The inhibitor, PP2, downregulates EMCV adsorption and entry. (**A**) A549 cells were plated in 96-well plates and treated with varying concentrations of PP2 (1.0, 2.5, 5.0, and 10.0 µM) for 24 h, with three replicate wells for each concentration. DMSO and blank control groups were also included. Cell viability was evaluated using a CCK-8 kit. (**B**) A549 cells were pre-treated with 5 µM PP2 for 1 h, with a DMSO control group for comparison. EMCV (at an MOI of 0.00001) was then inoculated into the cells, and samples were collected at 12, 24, and 36 hpi for the extraction of viral RNA. TaqMan probe-based RT-qPCR was performed to detect EMCV 3D gene copies. (**C and D**) Following treatment with 5 µM PP2, EMCV (at an MOI of 3) was inoculated onto A549 cells. The relative levels of EMCV 3D mRNA were measured in the attachment and entry assays. All genes detected by RT-qPCR were normalized to GAPDH expression. Statistical significance was indicated as follows: ***P < 0.001, *P < 0.05.

To investigate whether the phosphorylation of Fyn influences EMCV replication, A549 cells were treated with the Fyn activity inhibitor PP2 (24) and subjected to EMCV infection experiments. As PP2 concentrations exceeding 20 µM demonstrated cytotoxicity in prior studies (25), we evaluated its effects within a lower range (0–10 µM) using the CCK-8 assay kit in A549 cells. PP2 at concentrations of ≤5 µM exhibited no adverse effects on cell viability (Fig. 2A). Subsequently, PP2-treated A549 cells were infected with EMCV at an MOI of 0.00001 and harvested at 12, 24, and 36 hpi. As illustrated in Fig. 2B, EMCV genomic RNA levels in PP2-treated cells were significantly reduced at all time points compared to the dimethyl sulfoxide (DMSO) control group. Additionally, the relative mRNA levels of the EMCV 3D gene were assessed using RT-qPCR in experiments focusing on virus adsorption and internalization. The results demonstrated that the EMCV 3D mRNA levels in PP2-treated cells were significantly downregulated compared to those in the DMSO control group, both during virus adsorption and internalization (Fig. 2C and D). These findings suggest that the phosphorylation of Fyn is associated with EMCV replication and may primarily play a role in the viral invasion stage.

### Src kinase regulates EMCV replication and entry

**Fig 3:**
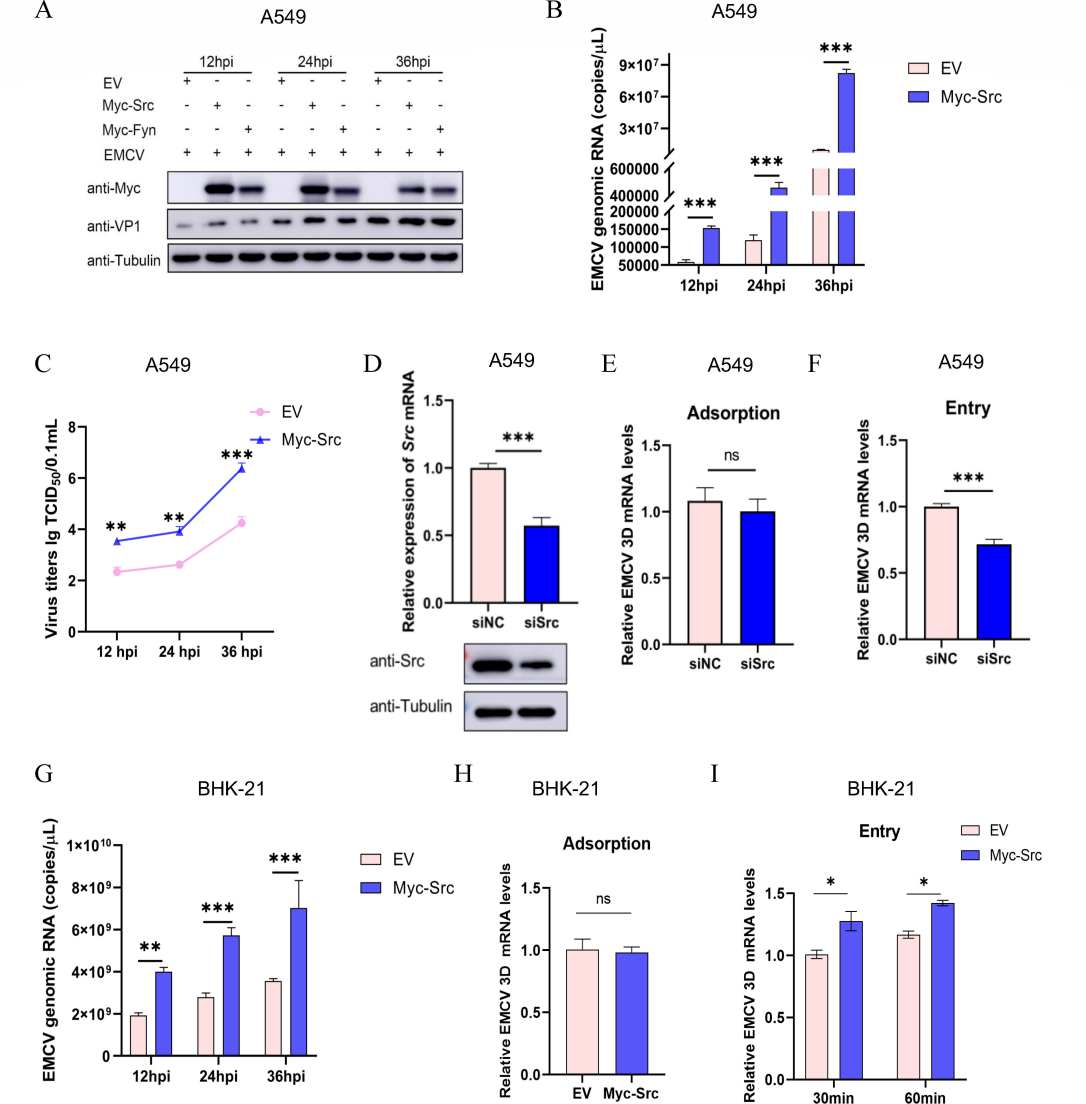
Overexpression of Src facilitates EMCV replication and entry. (**A**) Myc-tagged Src or Fyn plasmids were transfected into A549 cells, and an EV group was set up. At 24 h post-transfection, EMCV (at an MOI of 0.00001) was inoculated into the cells, and samples were collected at 12, 24, and 36 hpi for Western blotting detection of viral VP1 protein. (**B**) The mRNA levels of the EMCV 3D gene were detected by TaqMan probe-based RT-qPCR. (**C**) Virus titers were measured by TCID_50_. (**D**) Two siRNAs, siSrc-888 and siNC, were respectively transfected into A549 cells, and then Src expression was independently detected using Western blotting (bottom panel) and RT-qPCR (top panel). The adsorption (**E**) and internalization (**F**) experiments were performed in siRNA-treated A549 cells. Cells were subjected to viral mRNA detection 30 min following viral internalization. (**G**) The experimental procedures outlined in panel **A** were replicated in BHK-21 cells, with viral genomic copies quantified by TaqMan probe-based RT-qPCR. (**H and I**) BHK-21 cells transfected with Myc-Src (or EV) were incubated with EMCV (at an MOI of 3) to conduct adsorption and internalization experiments. The treated cells were collected, and total RNA was extracted. Subsequently, the relative levels of EMCV 3D mRNA were measured by RT-qPCR. All genes detected by RT-qPCR were normalized to GAPDH expression. ***P < 0.001, **P < 0.01, *P < 0.05. ns, no significant difference.

To explore Src’s role in EMCV infection, A549 cells were transfected with Myc-tagged Src plasmid or EV control. Twenty-four hours post-transfection, cells were infected with EMCV and harvested at 12, 24, and 36 hpi to evaluate viral replication and proliferation. Overexpression of Src significantly enhanced EMCV VP1 protein expression at three time points compared to EV groups (Fig. 3A), with Fyn also showing a positive regulatory effect (Fig. 3A). Subsequently, the quantities of EMCV genome copies and viral titer were determined through TaqMan probe-based RT-qPCR and 50% tissue culture infectious dose (TCID_50_) assays, respectively. As illustrated in Fig. 3B and C, a significant elevation in both EMCV genomic copies and viral titer was observed in Src-overexpressing A549 cells relative to the EV control groups at 12, 24, and 36 hpi. These findings adequately demonstrate that overexpression of Src enhances the replication and proliferation of EMCV in A549 cells. Furthermore, upon successfully knocking down Src expression (Fig. 3D), our findings revealed that, in comparison to the siNC control group, the EMCV 3D mRNA level remained unaffected in the viral adsorption experiment (Fig. 3E). However, it was notably downregulated in the internalization assay when contrasted with the siNC control group (Fig. 3F).

As the BHK-21 cell line is a commonly susceptible cell line for EMCV and is frequently used for virus propagation in laboratories, exploring the entry mechanism of EMCV into BHK-21 cells holds greater biological significance. Multiple studies have demonstrated that Src serves as an activator of caveolin-mediated endocytosis (CavME) (26). First, we found the EMCV genomic copies in BHK-21 cells also exhibited a marked upregulation in the Src overexpressed group (Fig. 3G). Furthermore, to ascertain whether Src plays a pivotal role in the entry of EMCV into BHK-21 cells, we conducted a series of experiments focusing on virus adsorption and internalization and analyzed the relative abundance of EMCV 3D mRNA in BHK-21 cells overexpressing Src. As illustrated in Fig. 3H, when compared to the EV control group, Src overexpression did not exert a significant impact on EMCV adsorption. However, it significantly facilitated virus entry into the cells, as evidenced by the data presented in Fig. 3I.

### The phosphorylation of Src plays a pivotal role in the replication and internalization of EMCV

**Fig 4:**
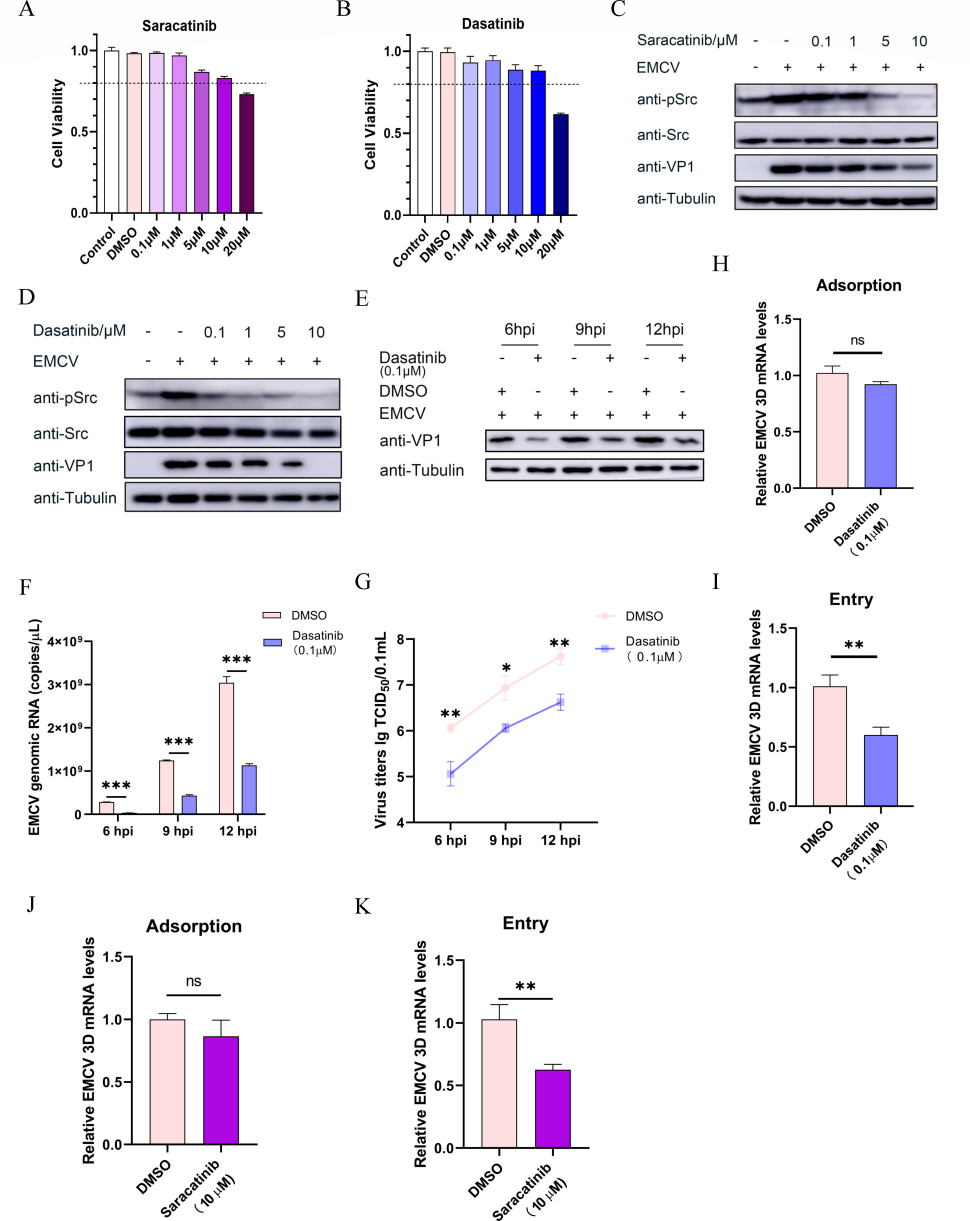
The phosphorylated Src kinase facilitates the replication and entry of EMCV into BHK-21 cells. (**A and B**) BHK-21 cells were cultivated in 96-well plates and incubated with various concentrations of inhibitors (saracatinib and dasatinib) for 24 h, with three replicate wells for each concentration gradient. DMSO and blank control groups were also established. Cell viability was assessed using a cell viability assay kit. (**C and D**) BHK-21 cells were pre-treated with different concentration gradients of saracatinib (0.1, 1.0, 5.0, and 10.0 µM) or dasatinib (0.1, 1.0, 5.0, and 10.0 µM) for 2 h, followed by inoculation with EMCV at an MOI of 1. Samples were collected at 6 hpi for the detection of src, phosphorylated Src (pSrc), and VP1 proteins using specific antibodies. (**E**) BHK-21 cells were pre-treated with dasatinib (0.1 µM) for 2 h, then inoculated with EMCV at an MOI of 0.1. Samples were collected at 6, 9, and 12 hpi for Western blot analysis of VP1 protein expression. (**F**) The mRNA level of the EMCV 3D gene was detected by TaqMan probe-based RT-qPCR. (**G**) Virus titers in the treated BHK-21 cells were measured by the TCID_50_ method. The relative expression level of EMCV 3d mRNA was detected by RT-qPCR in BHK-21 cells pre-treated with dasatinib (0.1 µM) (**H and I**) or saracatinib (10.0 µM) (**J and K**) from EMCV attachment and entry experiments. All genes detected by RT-qPCR were normalized to GAPDH expression levels. Statistical significance was indicated as follows: ***P < 0.001, **P < 0.01, *P < 0.05. ns, no significant difference.

Src and Src family tyrosine kinases are crucial regulatory enzymes involved in modulating cellular processes including differentiation, motility, proliferation, and survival. Src kinases feature two principal phosphorylation sites: an autoactivated tyrosine residue at position 416 (Y416) generated through autophosphorylation and an inhibitory phosphorylation site at position 527 (Y527) mediated by C-terminal Src kinase and its homolog, Chk (27). Both saracatinib and dasatinib, Src inhibitors, effectively inhibit tyrosine phosphorylation of Src at the human Y419 or murine Y416 site (18, 19). The 50% inhibitory concentration (IC_50_) values of dasatinib and saracatinib exhibit considerable variation across different cell lines. For example, dasatinib shows IC_50_ values ranging from 0.7 to 14.2 µM across nine hepatocellular carcinoma cell lines (28), while saracatinib exhibits values between 10.92 and 26.64 µM in various prostate cancer cell lines (29). As the effects of these two inhibitors on BHK-21 cells have not been reported, we first assessed the impact of dasatinib or saracatinib (at concentrations ranging from 0.1 to 20.0 µM) on BHK-21 cell proliferation using a CCK-8 kit and found that the maximum non-toxic concentration for both inhibitors was 10 µM (Fig. 4A and B). To investigate the relationship between Src phosphorylation and EMCV infection, we examined the regulatory effects of inhibitors at different concentrations on EMCV-activated Src phosphorylation and viral protein VP1 expression by Western blotting. Notably, Western blot analysis revealed a dose-dependent decrease in both Src phosphorylation and VP1 expression in inhibitor-treated cells relative to DMSO-treated controls (Fig. 4C and D). Here, we also observed that under the same concentration, dasatinib exhibited significantly stronger inhibitory effects on EMCV VP1 protein expression compared to saracatinib. Even at a low concentration (0.1 µM), it showed notable inhibitory activity (Fig. 4D), which is consistent with the findings in reference 30. Therefore, we chose 0.1 µM dasatinib for subsequent experiments.

To further elucidate the impact of Src inhibitors on EMCV propagation, BHK-21 cells were pre-treated with dasatinib. Subsequently, samples were collected at various time points (6, 9, and 12 hpi) to analyze the replication and proliferation of EMCV. The results indicated that dasatinib treatment significantly downregulated the VP1 protein expression compared to the DMSO control at all three time points (Fig. 4E). Consequently, we investigated the regulatory effects of dasatinib on EMCV viral copies and virus titer. Our findings revealed that Src phosphorylation inhibition markedly decreased EMCV replication and proliferation compared to the DMSO group (Fig. 4F and G). Additionally, dasatinib treatment had no significant impact on EMCV adsorption (Fig. 4H) but effectively inhibited virus internalization (Fig. 4I). Moreover, saracatinib treatment exhibited similar results (Fig. 4J and K). These results imply that the phosphorylation of the tyrosine residue at position 419 (Y419) within Src plays a crucial role in the entry of EMCV into BHK-21 cells.

### Ezrin plays a key role in EMCV replication and entry

As a significant constituent of the ERM protein family, Ezrin fulfills its distinctive functions through its binding to actin, thereby playing a vital role in cellular migration and endocytosis processes (31). Prior studies have documented the indispensable role of Ezrin in facilitating the entry of rhinovirus, severe acute respiratory syndrome coronavirus, and JEV into their respective target cells (15, 32, 33). In this investigation, our objective is to elucidate whether Ezrin similarly serves a crucial function in the entry of EMCV into BHK-21 cells.

**Fig 5:**
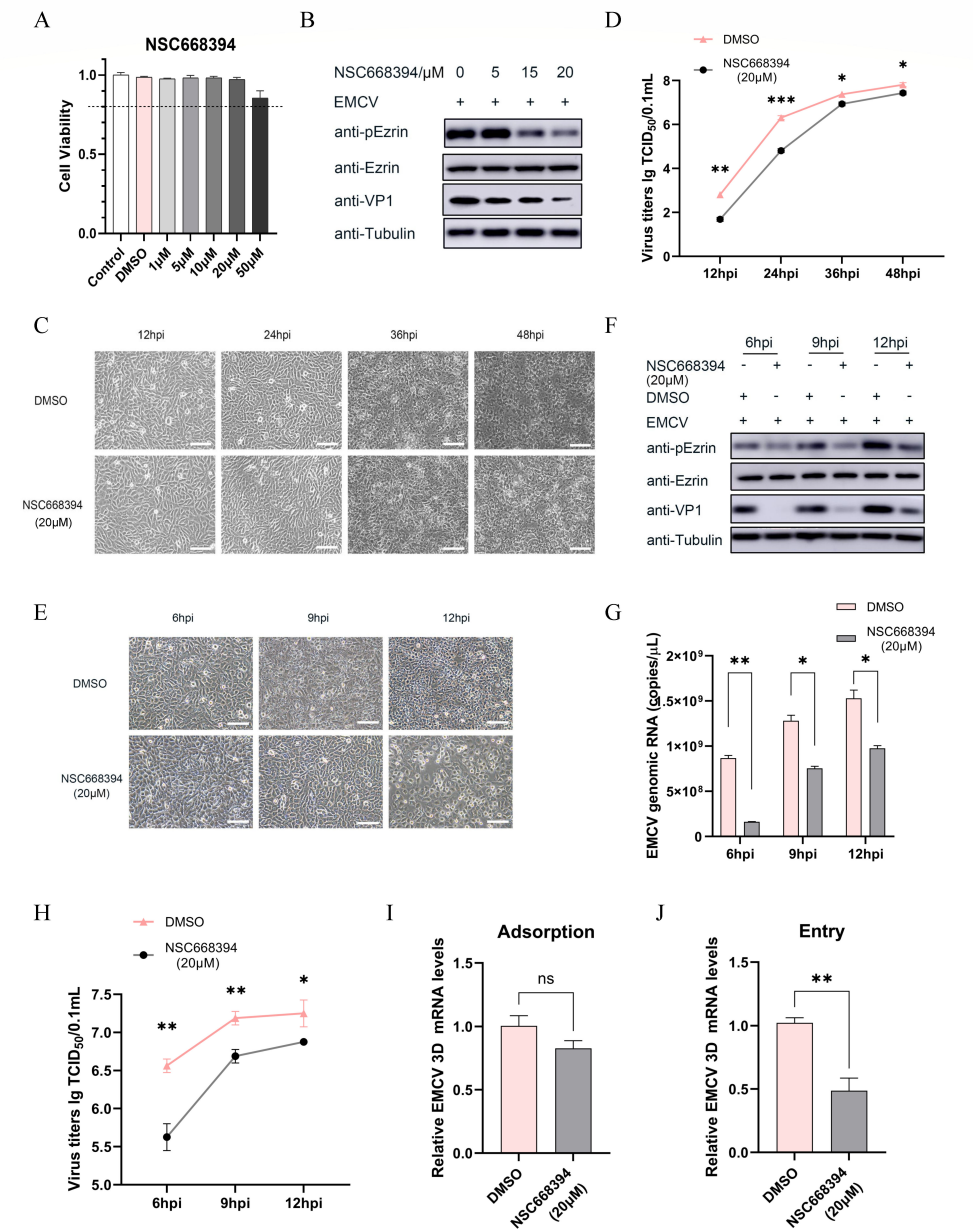
Ezrin regulates the replication, proliferation, and internalization of EMCV. (**A**) A CCK-8 assay kit was utilized to assess the cell viability of BHK-21 cells treated with various concentrations (1, 5, 10, 20, and 50 µM) of NSC663894. Controls included DMSO and a blank control group. (**B**) BHK-21 cells were pre-treated with different concentrations (0, 5, 15, and 20 µM) of NSC663894 for 1 h prior to inoculation with EMCV (MOI of 1). Samples were collected 6 hpi, and Western blotting was performed using specific antibodies to detect proteins such as VP1, Ezrin, and phosphorylated Ezrin, with tubulin serving as an internal control. (**C**) BHK-21 cells were pre-treated with the maximum working concentration (20 µM) of NSC663894 for 1 h, with a DMSO control group also included. The cells were then inoculated with a low dose of EMCV (MOI of 0.000001), and CPE was observed and photographed under a microscope at 12, 24, 36, and 48 hpi (scale bar, 100 µm). (**D**) The aforementioned treated BHK-21 cells were collected, and viral titers were determined using the TCID_50_ method. (**E**) Higher-dose EMCV (MOI of 0.1) was inoculated into BHK-21 cells, and CPEs were observed under a microscope at different time points (6, 9, and 12 hpi) (scale bar, 100 µm). (**F**) The collected cells from the above treatment were analyzed by immunoblotting to detect the expression levels of indicated proteins, such as EMCV VP1, Ezrin, and phosphorylated Ezrin. (**G**) Viral RNA was extracted to quantify the virus copy number by TaqMan probe-based RT-qPCR. (**H**) Viral titers were determined using the TCID_50_ method. (**I and J**) RT-qPCR was employed to detect the relative expression level of EMCV 3D mRNA in EMCV adsorption and entry experiments after pre-treating cells with NSC663894. All RT-qPCR gene levels were normalized to GAPDH. ***P < 0.001, **P < 0.01, *P < 0.05. ns, no significant difference.

First, we investigated the role of Ezrin in the replication process of EMCV. NSC663284 (IC_50_ = 8.1 µM) is a quinolone derivative that binds Ezrin and specifically inhibits phosphorylation of its Thr567 residue. This phosphorylation event is required for Ezrin activation and subsequent F-actin binding. Previous studies on JEV entry mechanisms demonstrated that a high concentration (40 µM) of NSC668394 significantly disrupts viral internalization (15). Therefore, we first assessed cytotoxicity of NSC663894 (1–50 μM) in BHK-21 cells using the CCK-8 assay. Cell viability remained >80% across 1–50 μM concentrations (Fig. 5A). Next, to demonstrate the impact of NSC663894 on EMCV replication and proliferation, BHK-21 cells were pre-treated with a gradient of concentrations (0, 5, 15, and 20 µM) of NSC663894 for 1 h prior to virus inoculation. The results of the western blot analysis revealed that, compared to the DMSO control, NSC663894 treatment significantly reduced both the phosphorylation level of Ezrin and the expression level of the viral protein VP1, exhibiting a clear dose-dependent relationship, as illustrated in Fig. 5B. To further elucidate the effect of NSC663894 on EMCV replication and propagation, BHK-21 cells were inoculated with a virus at a low MOI. The CPE was observed at 12, 24, 36, and 48 hpi. Subsequently, the treated cells were collected for virus titer detection. As shown in Fig. 5C, microscopic observation demonstrated that the NSC663894-treated group exhibited a less severe CPE at 36 and 48 hpi compared to the DMSO group. This finding was further corroborated by the viral propagation curve presented in Fig. 5D.

Subsequently, to investigate the inhibitory impact of NSC663894 on EMCV at a higher viral dose, BHK-21 cells were inoculated with EMCV at an MOI of 0.1. Microscopic examination revealed that the DMSO control group exhibited CPE as early as 9 hpi, whereas the NSC663894-treated group delayed the onset of CPE until 12 hpi, and the CPE observed was relatively mild (Fig. 5E). Furthermore, our findings indicated that NSC663894 downregulated the expression of EMCV VP1, which was accompanied by the inhibition of Ezrin phosphorylation (Fig. 5F). Additionally, the number of EMCV genome copies and virus titer were quantified at 6, 9, and 12 hpi (Fig. 5G and H).

To further elucidate the role of Ezrin in the internalization process of EMCV, we conducted cell adsorption and invasion experiments with NSC663894 treatment. In the virus adsorption experiment, no significant difference in the expression level of the EMCV 3D gene was observed between the DMSO-treated and NSC663894-treated groups (Fig. 5I). However, in the virus invasion experiment, a notable downregulation of viral gene expression was evident in the NSC663894-treated group (Fig. 5J). These findings suggest that the phosphorylation status of Ezrin plays a pivotal role in the entry of EMCV into cells. Consequently, NSC663894 holds potential as a promising anti-EMCV therapeutic agent.

### Cav-1 is essential for EMCV entry into cells

**Fig 6:**
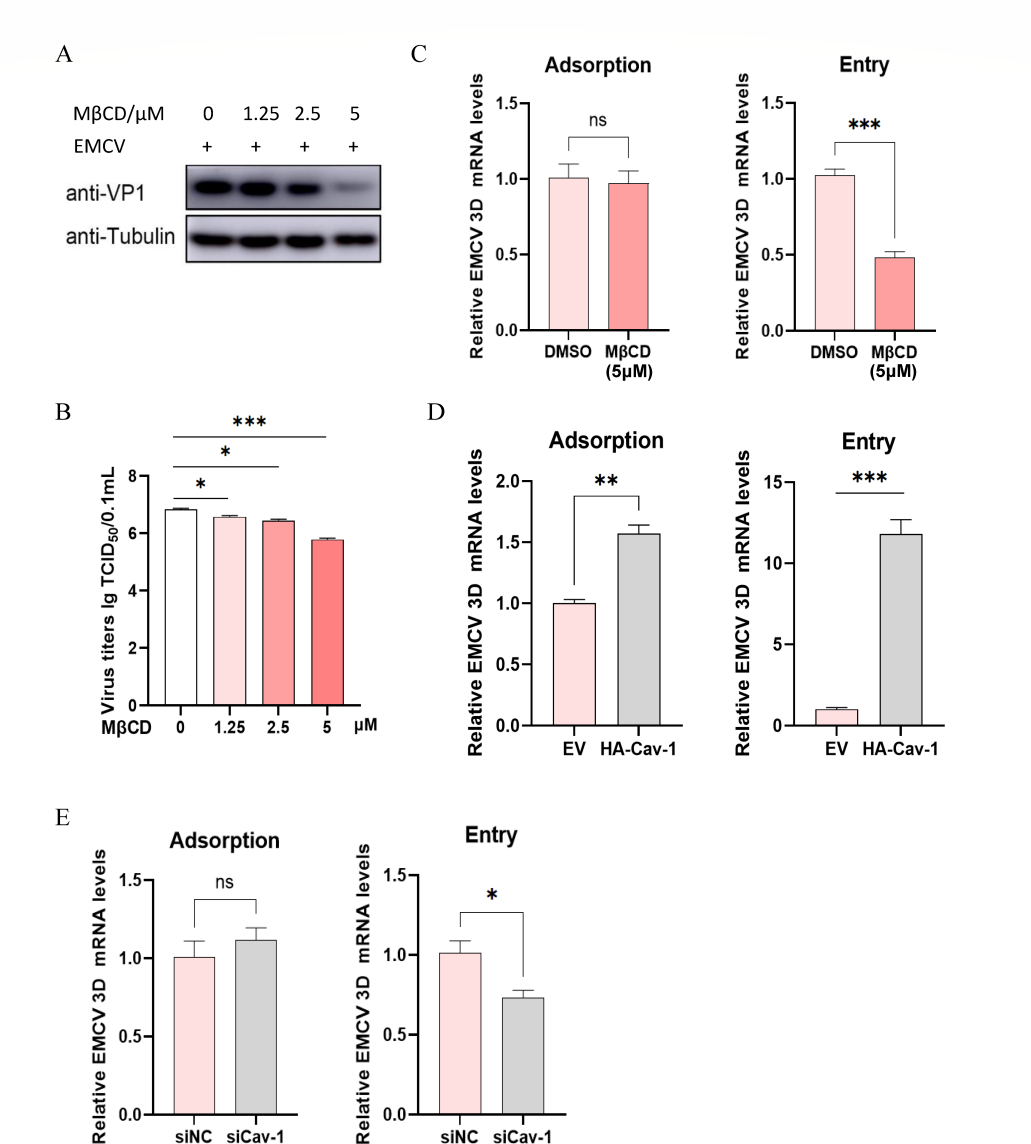
Cav-1 is indispensable for the entry of EMCV into BHK-21 cells. (**A**) BHK-21 cells were pre-treated with various concentrations (0, 1.25, 2.5, and 5.0 µM) of MβCD for 1 h, followed by inoculation with EMCV at an MOI of 1. Samples were collected 6 h post-infection, and the VP1 protein was detected by Western blotting, with tubulin serving as the internal control protein. (**B**) BHK-21 cells and its supernatants that have undergone the same aforementioned (**A**) treatment, followed by repeated freeze-thaw cycles, were subjected to TCID_50_ assay. (**C**) BHK-21 cells were pre-treated with MβCD (5 µM) for 1 h, with DMSO serving as a control. Subsequently, EMCV was inoculated at an MOI of 3 to perform adsorption and internalization experiments, and the relative expression level of the EMCV 3D gene was detected by RT-qPCR. (**D**) BHK-21 cells were transfected with the HA-Cav-1 recombinant plasmid, and the relative expression level of the EMCV 3D gene was assessed by RT-qPCR in both adsorption and internalization experiments. (**E**) Small interfering RNA (siCav-1 or siNC) was transfected into BHK-21 cells. Following 24 h of transfection, EMCV (MOI = 3) was used to perform adsorption and internalization experiments, and the relative expression level of the EMCV 3D gene was detected by RT-qPCR. The 3D gene expression levels detected by RT-qPCR were normalized to GAPDH. Statistical significance was determined as follows: ***P < 0.001, **P < 0.01, *P < 0.05. ns, no significant difference.

Our previous laboratory studies have established that EMCV enters BHK-21 cells through CavME, with Cav-1 playing a pivotal role in EMCV infection (10). To further validate this, we pre-treated BHK-21 cells with varying concentrations of the CavME inhibitor, MβCD, prior to EMCV inoculation. Both the detection of viral VP1 protein and virus titer confirmed that EMCV replication was significantly downregulated in the MβCD-treated group compared to the control group, displaying a clear dose-dependent relationship (Fig. 6A and B). In adsorption and internalization experiments, we observed a significant decrease in the relative mRNA level of the EMCV 3D gene in the MβCD-treated group compared to the DMSO control specifically during the internalization stage but not during the adsorption stage (Fig. 6C). These findings strongly support the notion that EMCV entry into BHK-21 cells is dependent on CavME.

Furthermore, when BHK-21 cells were transfected with the recombinant plasmid HA-Cav-1, both EMCV internalization and adsorption levels were significantly increased (Fig. 6D). Conversely, when BHK-21 cells were transfected with specific siRNA targeting Cav-1 and subjected to adsorption and internalization experiments, the results depicted in Fig. 6E showed that viral internalization levels were specifically and significantly reduced compared to the siNC control group after Cav-1 expression was knocked down. These results unequivocally confirm that Cav-1 is essential for EMCV entry into cells.

### Src, Ezrin, and Cav-1 are crucial for EMCV entry

**Fig 7:**
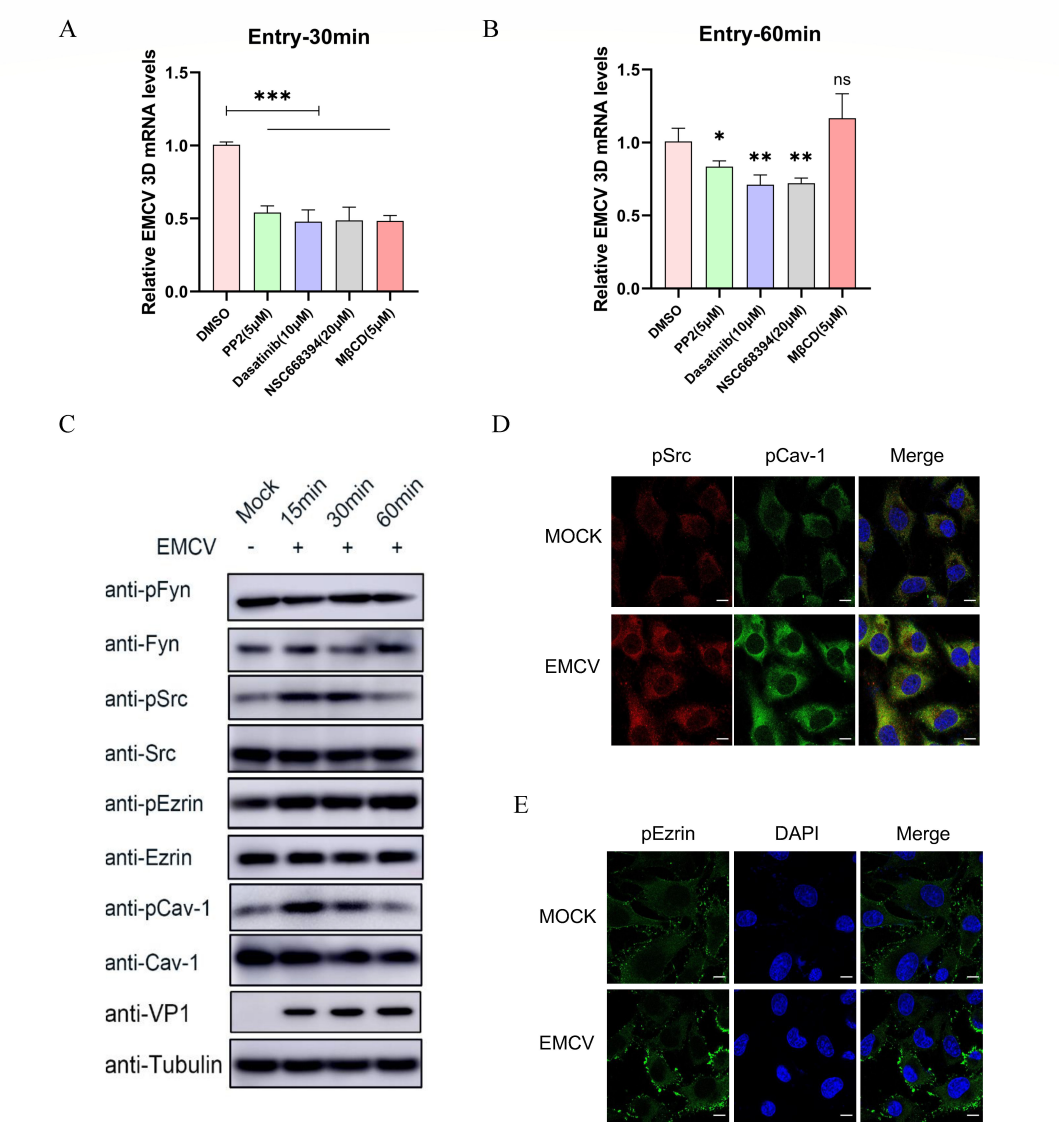
Expression and phosphorylation of Src, Ezrin, and Cav-1 during EMCV internalization. (**A** and **B**) Cells were pre-treated with various inhibitors, PP2 (5 µM), dasatinib (10 µM), NSC663894 (20 µM), and MβCD (5 µM) prior to inoculation with EMCV (3 MOI) in BHK-21 cells. Following a 1 h adsorption period at 4°C, the cells were incubated at 37°C. At 30 or 60 min post-incubation, the cells were trypsinized, collected, and subjected to total RNA extraction. RT-qPCR was employed to quantify the relative expression of the EMCV 3D mRNA. (**C**) BHK-21 cells were plated in six-well plates and infected with EMCV (3 MOI) once the cell density reached 90%. Samples were collected at 0, 15, 30, and 60 min post-infection. Western blotting was utilized to assess the expression levels of VP1, Fyn, Src, Ezrin, Cav-1, and their phosphorylated forms, with Tubulin serving as a reference protein. (**D**) BHK-21 cells were plated on cell slides and infected with EMCV (3 MOI). After a 15 min incubation period, the cells were fixed and immunostained to detect pSrc and pCav-1. The nuclei were stained with DAPI (blue). The fluorescence signals were visualized under a confocal microscope (LSM 900 with Airyscan 2), with a scale bar of 10 µm. (**E**) Localization of pEzrin in MOCK- and EMCV-infected cells: the localization of pEzrin in both MOCK- and EMCV-infected BHK-21 cells was detected using the same immunostaining method described above. Statistical significance was indicated as follows: *** *P* < 0.001, ** *P* < 0.01, * *P* < 0.05, and ns indicates no significant difference.

Our results have demonstrated the significance of the proteins Fyn, Src, Ezrin, and Cav-1 in the internalization process of EMCV. Specifically, BHK-21 cells were treated with specific inhibitors targeting these four proteins: PP2 for Fyn, dasatinib for Src, NSC663894 for Ezrin, and MβCD for Cav-1. Following this treatment, EMCV internalization experiments were conducted. The detection of relative EMCV 3D mRNA levels revealed that, compared to the DMSO control group, EMCV entry was significantly downregulated (*P* < 0.001) in all four inhibitor-treated groups at 30 min post-internalization (Fig. 7A). Notably, when the internalization time was extended to 60 min, the inhibitory effect observed in the MβCD-treated group diminished (*P* > 0.05) (Fig. 7B). This suggests that EMCV internalization is more active and sensitive to inhibition at the earlier 30 min time point compared to the later 60 min time point. These findings underscore the crucial roles of Src, Ezrin, and Cav-1 in facilitating EMCV entry into cells.

To elucidate the regulatory effects of EMCV on Fyn, Src, Ezrin, and Cav-1 during its internalization process, an EMCV internalization experiment was conducted, with samples collected at various time points (0, 15, 30, and 60 min). Western blot analysis was performed to assess the protein expression levels of Fyn, Src, Ezrin, Cav-1, and their phosphorylated forms. As depicted in Fig. 7C, as the internalization progresses, the level of EMCV VP1 protein gradually increases. Concurrently, the expression of the phosphorylated forms of Src (pSrc), Ezrin (pEzrin), and Cav-1 (pCav-1) is significantly upregulated at 15 and 30 min post-EMCV infection, reaching a peak at 15 min. By 60 min, the expression of pSrc and pCav-1 nearly returns to pre-infection levels, indicating a trend of initial upregulation followed by downregulation in the activation of these two proteins during EMCV internalization. Notably, there are no significant changes in the total protein levels of Src, Ezrin, and Cav-1 across the three time points. Surprisingly, compared to the uninfected group, neither Fyn nor its phosphorylated form exhibits significant changes during EMCV internalization and, therefore, will not be discussed further in this context (Fig. 7C). These findings suggest that the phosphorylation status of Src, Ezrin, and Cav-1 may play a crucial role in facilitating EMCV internalization.

To further elucidate the effects of EMCV infection on the activation and intracellular localization of Src, Ezrin, and Cav-1, BHK-21 cells were infected with EMCV for 15 min, followed by indirect immunofluorescence assays (IFAs). As illustrated in Fig. 7D, compared to the MOCK group, the fluorescence signals of pSrc and pCav-1 were significantly enhanced in the EMCV-infected group, with clear colocalization observed. Furthermore, it was also found that, compared to the MOCK group, the fluorescence signal of pEzrin, bound to a FITC-conjugated secondary antibody, was enhanced following EMCV infection (Fig. 7E). This may be related to the activation of Ezrin by EMCV, promoting its interaction with actin and thereby facilitating virus internalization (31). Thus, the involvement of Src, Ezrin, and Cav-1 in the process of EMCV internalization has been further substantiated.

### pSrc, pEzrin, and pCav-1 are sequentially activated during EMCV infection

**Fig 8:**
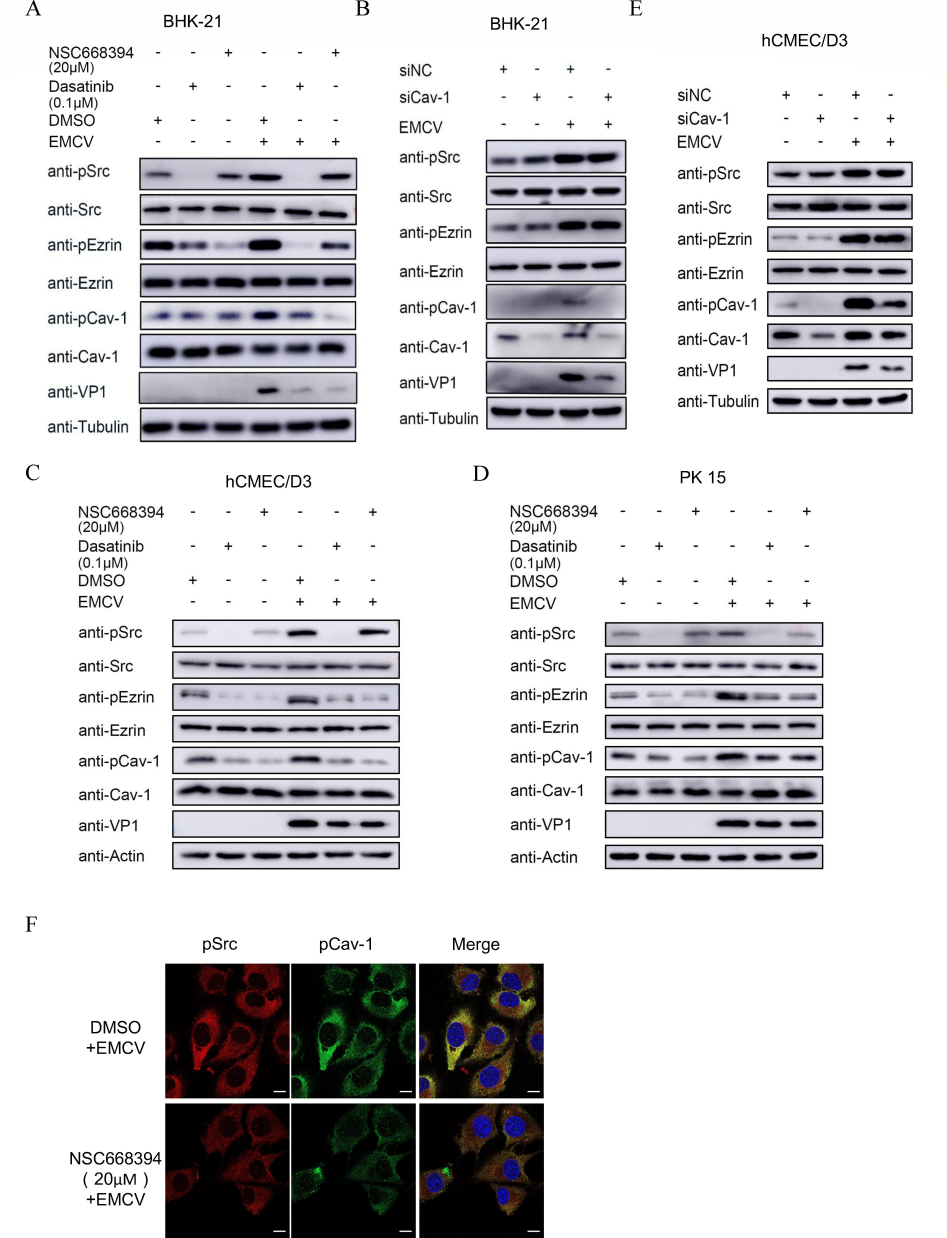
Regulatory relationships among Src, Ezrin, and Cav-1. (**A**) BHK-21 cells were pre-treated with 10 µM dasatinib or 20 µM NSC663894 for 2 h, respectively, and then mock-infected or infected with EMCV at an MOI of 3 for 30 min. DMSO was served as a control. Following this, the cells were either mock-infected or EMCV-infected at an MOI of 3 for 30 min. Protein expression levels of EMCV VP1, Src/pSrc, Ezrin/pEzrin, Cav-1/pCav-1 were detected by Western blotting using specific antibodies. (**B**) BHK-21 cells were transfected with siCav-1 (or siNC) using Lipo3000 transfection reagent. After 24 h of incubation, the cells were inoculated with EMCV at an MOI of 3 for 30 min before being collected for Western blot analysis. Experiments similar to those described in panel **A** were conducted on hCMEC/D3 cells (**C**) and PK15 cells (**D**) using 0.1 µM dasatinib or 20 µM NSC663894, respectively. The viral inoculation dose was an MOI of 10 for hCMEC/D3 cells and an MOI of 3 for PK15 cells. (**E**) hCMEC/D3 cells were transfected with siCav-1 (or siNC) and inoculated with EMCV at an MOI of 10 for 30 min. The levels of the related proteins were detected by Western blotting. (**F**) BHK-21 cells were plated on coverslips and pre-treated with 20 µM NSC663894 for 1 h when the cell confluence reached 50%. The cells were then infected with EMCV at an MOI of 3 for 30 min. Following fixation, pSrc and pCav-1 in the treated cells were labeled using specific antibodies and fluorescently tagged secondary antibodies. Nuclei were stained with DAPI dye. The scale bar represents 10 µm.

To investigate the potential mutual regulatory interactions between pSrc, pEzrin, and pCav-1 during EMCV internalization, we employed western blotting to assess the levels of pSrc, pEzrin, and pCav-1 in both EMCV-infected and MOCK-infected BHK-21 cells following treatment with dasatinib or NSC663894, or siCav transfection. Notably, in comparison to the DMSO control, the Dasatinib-treated groups exhibited significant downregulation of pSrc, pEzrin, and pCav-1 levels, with this effect being more pronounced under EMCV infection conditions (Fig. 8A). Consistent with expectations, NSC663894 had no significant impact on Src or pSrc levels in either infected or uninfected BHK-21 cells when compared to the DMSO group (Fig. 8A). Additionally, both inhibitors led to a marked reduction in the levels of the viral protein VP1 compared to the DMSO control group. Importantly, both inhibitors significantly suppressed the phosphorylation of Cav-1, suggesting a sequential regulatory relationship among pSrc, pEzrin, and pCav-1 in EMCV-infected BHK-21 cells. We further verified this through Cav-1 knockout experiments. Specifically, Fig. 8B demonstrates that siRNA targeting Cav-1 significantly reduced Cav-1 and pCav-1 protein levels in BHK-21 cells, accompanied by a downregulation of VP1 protein and an inhibition of EMCV-activated phosphorylation of Cav-1. Meanwhile, compared to the siNC group, there were no significant changes in the expression levels of Src/pSrc and Ezrin/pEzrin in the siCav-1 group (Fig. 8B).

To ascertain whether similar cascade reactions occur during EMCV invasion of other cell types, we selected two additional cell lines (hCMEC/D3 and PK15) for verification. Following the separate treatment of hCMEC/D3 (or PK15) cells with two distinct inhibitors (dasatinib and NSC663894), they were subjected to EMCV inoculation for viral internalization experiments. The target proteins were detected as described in Fig. 8A, and comparable results were observed. Specifically, dasatinib led to the downregulation of protein levels of pSrc, pEzrin, and pCav-1, whereas NSC663894 downregulated the protein levels of pEzrin and pCav-1 (Fig. 8C and D). Both inhibitors resulted in the downregulation of EMCV invasion into hCMEC/D3 and PK15 cells (Fig. 8C and D). Furthermore, in alignment with the observations in BHK21 cells, transfection of hCMEC/D3 cells with siCav-1 exclusively downregulated the expression of Cav-1 and pCav-1, as well as viral protein VP1 (Fig. 8E). These findings collectively imply that EMCV invasion into susceptible cells triggers the sequential activation of pSrc/pEzrin/pCav-1, thereby orchestrating viral invasion.

To further substantiate the relationship between pSrc, pEzrin, and pCav-1, we performed IFA to assess the expression and localization of pSrc and pCav-1 during EMCV entry into BHK-21 cells treated with the pEzrin inhibitor NSC663894 (DMSO as control). Notably, NSC663894 treatment diminished the green fluorescent signal of pCav-1 compared to DMSO controls (Fig. 8F). Collectively, these findings suggest a sequential activation cascade involving pSrc, pEzrin, and pCav-1 during the internalization process of EMCV.

### ICAM-1 regulates on the Src and Src-Ezrin-Cav-1 pathway

**Fig 9:**
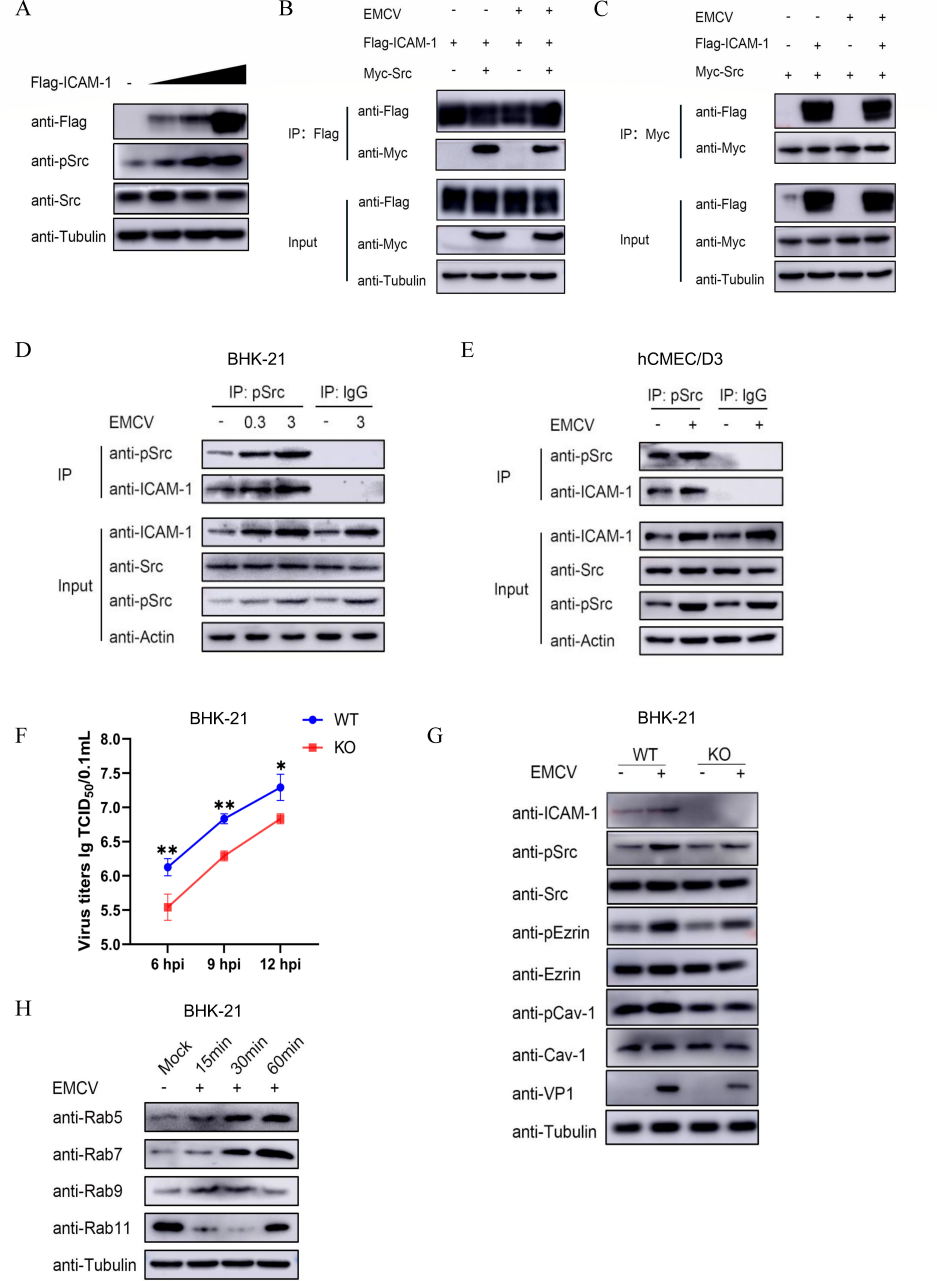
Regulation of ICAM-1 on the Src and Src-Ezrin-Cav-1 pathway. (**A**) Different concentrations (0.5, 1.0, and 2.0 µg) of the Flag-ICAM-1 plasmid were transfected into BHK-21 cells. Cells were collected, and the expressions of Flag-ICAM-1 and Src/pSrc were analyzed by Western blotting. (**B** and **C**) Two recombinant plasmids, Flag-ICAM-1 and Myc-Src, were co-transfected into HEK 293T cells, with an empty vector serving as the control. After 36 h of transfection, the cells were inoculated with EMCV at an MOI of 3. Thirty min later, the cells were collected and subjected to Co-IP analysis using agarose beads coated with Flag or Myc monoclonal antibodies. The immune precipitates were then analyzed by Western blotting. (**D**) Endogenous Co-IP was performed with BHK-21 cells infected with EMCV (MOI of 0, 0.3, or 3.0) for 30 min using a specific antibody against pSrc. (**E**) Endogenous Co-IP was performed with hCMEC/D3 cells infected with EMCV (MOI of 10), with a mock control for 30 min using a specific antibody against pSrc. (**F**) EMCV titration was measured after inoculation on EMCV (MOI of 0.1) in wild-type (WT) BHK-21 cell lines and ICAM-1 knockout (KO) BHK-21 cell lines. (G) EMCV was inoculated into WT BHK-21 cell lines and ICAM-1 KO BHK-21 cell lines at an MOI of 3 for 30 min, with mock controls. Western blot analysis was performed to detect changes in the expression of VP1, Src/pSrc, Ezrin/pEzrin, Cav-1/pCav-1. (H) BHK-21 cells with a cell density exceeding 90% were inoculated with EMCV at an MOI of 3. Cells were collected at 0, 15, 30, and 60 min post-infection, and immunoblotting analysis was performed to detect the expression of Rab5, Rab7, Rab9, and Rab11 following EMCV infection. Statistical significance was indicated as follows: ** *P* < 0.01, * *P* < 0.05.

ICAM-1, a member of the immunoglobulin superfamily, is located on the surface of endothelial and epithelial cells, fibroblasts, and specific hematopoietic cells (34, 35). It serves as a ligand for leukocyte integrins and a receptor for fibrinogen (36, 37), thereby facilitating the entry of diverse picornaviruses, such as Coxsackievirus A21, Enterovirus 71 (38), and rhinoviruses (39, 40). Recently, our team has uncovered enough evidence suggesting that ICAM-1 is implicated in the attachment and internalization of EMCV (Z. Y. Hou et al., unpublished data), prompting the hypothesis that ICAM-1 may exhibit a regulatory interplay with internalization-related proteins aforementioned. To verify the hypothesis, we transfected BHK-21 cells with different concentrations of the recombinant plasmid Flag-ICAM-1 and assessed Src activation. Results showed a dose-dependent increase in pSrc levels with rising Flag-ICAM-1 expression, while total Src remained unchanged (Fig. 9A). We then co-transfected the plasmids Myc-Src and Flag-ICAM-1 into HEK 293T cells and then performed co-immunoprecipitation (Co-IP) assay using Flag and Myc monoclonal antibodies to explore the potential interaction between ICAM-1 and Src. As shown in Fig. 9B and C, whether Myc or Flag antibody served as the bait, overexpressed Flag-ICAM-1 and Myc-Src exhibited interaction. However, no discrepancy in the interaction between these two recombinant proteins was observed irrespective of virus infection. Consequently, we delved deeper into the interaction between endogenous ICAM-1 and phosphorylated Src under EMCV infection conditions. Following the internalization assays of EMCV (at MOIs of 0.3 or 3.0) in BHK-21 cells, the cells were harvested and subjected to an IP assay using an antibody against pSrc. Western blot analysis revealed that as the MOI increased, the level of pSrc rose, along with an increase in its binding to ICAM-1 (Fig. 9D). Furthermore, we observed that in hCMEC/D3 cells, EMCV infection led to the upregulation of pSrc and enhanced its interaction with ICAM-1 (Fig. 9E).

To ascertain whether ICAM-1 regulates the Src-Ezrin-Cav-1 pathway via Src to jointly regulate EMCV internalization, we initially analyzed the viral titers in both wild-type (WT) BHK-21 cell lines and ICAM-1 KO BHK-21 cell lines following EMCV infection. We observed that the viral proliferation level was lower in the latter cell line at 6, 9 and 12 hpi, with a significant difference in the proliferation capability of EMCV between these two cell lines (Fig. 9F). Subsequently, we conducted an EMCV internalization assay in these two cell lines and analyzed the expression level of ICAM-1 as well as its impact on the virus-activated pSrc-pEzrin-pCav-1 pathway. As depicted in Fig. 9G, upon EMCV infection in the presence of ICAM-1, the expression levels of pSrc, pEzrin, and pCav-1 were significantly upregulated in WT BHK-21 cells compared to the uninfected control. Conversely, in the ICAM-1 KO BHK-21 cell line, the EMCV-induced upregulation of pSrc, pEzrin, and pCav-1 was markedly inhibited, accompanied by a reduction of VP1 protein (Fig. 9G). These findings suggest that ICAM-1 plays a crucial regulatory role in the activation of Src and the Src-Ezrin-Cav-1 pathway during EMCV entry into BHK-21 cells.

Rab proteins are small GTPases belonging to the Ras superfamily, also referred to as small G proteins. They regulate the dynamics of the vesicular network within the endocytic pathway, thereby controlling intracellular material transport (41, 42). Rab5 mediates the movement of newly internalized vesicles to early endosomes; Rab7 oversees early endosomal maturation and vesicle trafficking to lysosomes; Rab9 facilitates late endosome integration into the trans-Golgi network; and Rab11 is vital for vesicle recycling. These four types of Rab proteins have been repeatedly reported to be involved in the internalization process of viruses, such as classical swine fever virus (43) and porcine enteric alphacoronavirus (42). This study preliminarily identified the expression changes of Rab proteins during the internalization process of EMCV. As illustrated in Fig. 9H, a marked upregulation in the protein expression levels of Rab5 and Rab7 was observed, with Rab9 and Rab11 proteins exhibiting dynamic fluctuations. This finding suggests the involvement of Rabs proteins in the internalization process of EMCV, as they may mediate the transport of endocytic vesicles containing viral particles.

**Fig 10:**
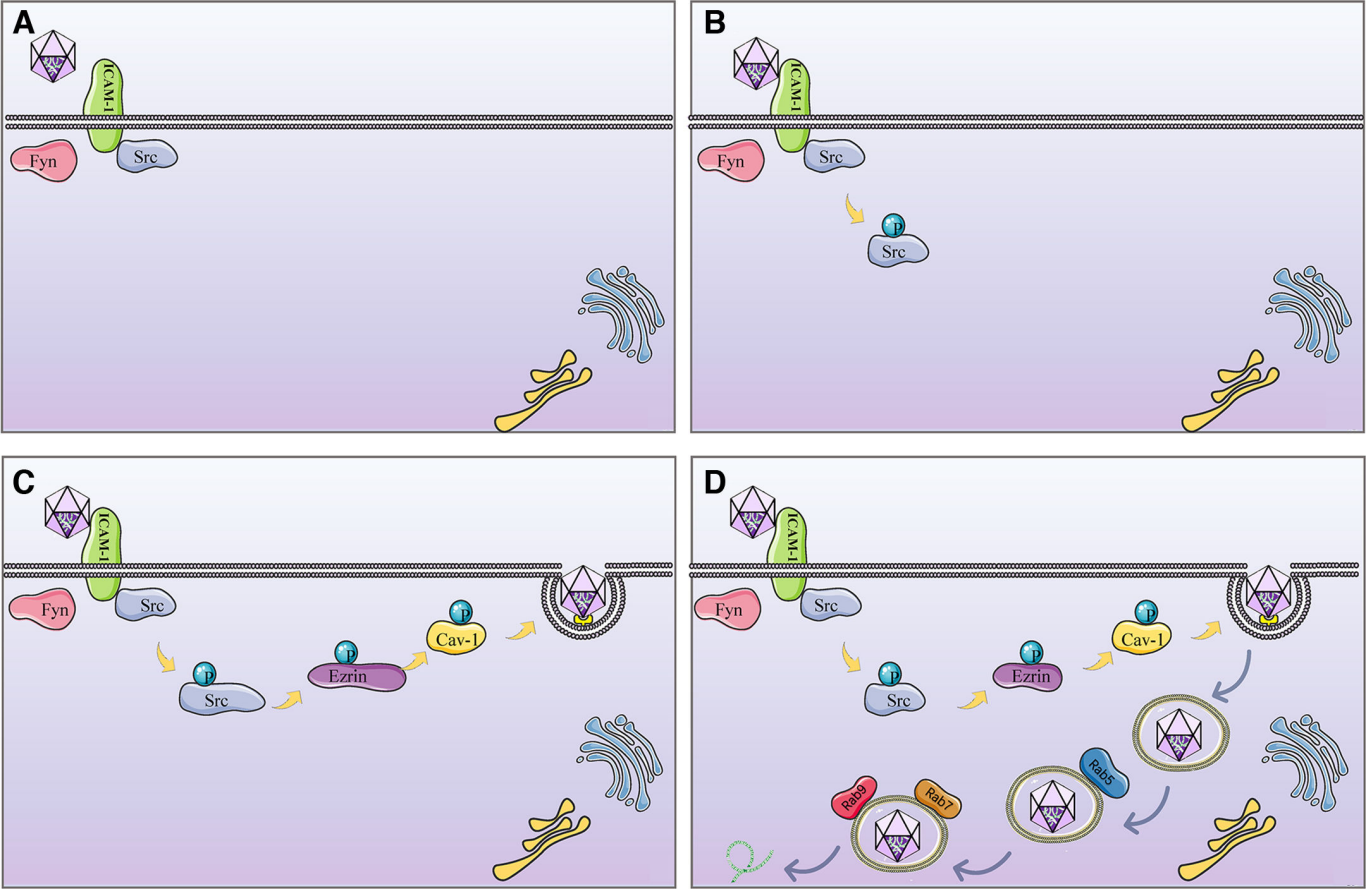
Entry of EMCV into BHK-21 cells occurs via the ICAM-1-mediated Src signaling pathway. (**A**) In BHK-21 cells, an interaction between ICAM-1 and Src exists prior to EMCV infection. (**B**) Upon infecting BHK-21 cells, EMCV recruits Src through its interaction with ICAM-1, resulting in the phosphorylation of Src. (**C**) The activated Src promotes the phosphorylation of Ezrin and Cav-1, thereby facilitating EMCV entry into host cells via CavME. (**D**) Once internalized, the viral particle is transported from early endosomes to late endosomes, ultimately completing its subsequent life-cycle activities.

In summary, during the invasion process of EMCV, there exists an internalization signaling pathway comprising ICAM-1-Src-Ezrin-Cav-1 (Fig. 10). Following internalization, EMCV is transported from early endosomes to late endosomes to continue its life cycle.

## DISCUSSION

Despite their relatively simple structure and composition, viruses exhibit intricate interactions with host cells. To gain cellular entry, viruses can mobilize hundreds of host proteins, facilitating the processes of adsorption and internalization (44). Viruses primarily invade host cells through two predominant mechanisms: membrane fusion and endocytosis. Notably, most viruses rely on receptor-mediated endocytosis, specifically, for cellular entry (45). Various viruses adopt distinct methods to enter host cells, primarily encompassing CME, CavME, and macropinocytosis. Previously, CME was regarded as the primary mode of viral cellular entry; however, recent discoveries have highlighted the significant role of CavME in viral invasion (45). Our research team has previously shown that EMCV enters host cells via CavME (10), yet the underlying molecular mechanism remains unclear. Herein, we report the identification of Fyn, Ezrin, Src, and Caveolin-1 as pivotal factors facilitating EMCV internalization. Furthermore, we have elucidated, for the first time, the existence of the ICAM-1-Src-Ezrin-Cav-1 pathway during viral entry into cells.

The phosphorylation activation facilitated by the non-receptor tyrosine kinase Src constitutes an early and crucial step in initiating the detachment of caveolae from the plasma membrane (26, 46). For instance, during the process of invading host cells, ARV stimulates the Src and p38 mitogen-activated protein kinase (MAPK) pathways, thereby promoting the formation of endocytic vesicles and facilitating viral entry (13, 47). Upon binding to receptors during invasion, NDV triggers Src activation, which mediates multiple signaling cascades that promote actin rearrangement and coordinates viral entry (14). After JEV infection, Src becomes activated, resulting in the phosphorylation of Ezrin in the cytoplasm and the formation of the Src/Ezrin/Caveolin-1 complex, thereby facilitating viral invasion (15). In summary, Src plays a crucial role in facilitating the entry process of various viruses into cells. Previous studies revealed that EMCV infection induces rapid upregulation of Src phosphorylation in macrophages during early infection. This subsequently activates the MAPK/p38 pathway, which regulates EMCV-induced COX-2 expression, demonstrating that Src family kinase (SFK) activation plays a positive regulatory role in inflammatory gene expression during viral infection of macrophages (RAW264.7) (48). However, whether Src participates in EMCV internalization remains unreported. In this study, we identified that the overexpression of Src kinase and its family member Fyn kinase individually enhanced the replication and proliferation of EMCV. The treatment with specific siRNA and inhibitors (saracatinib and dasatinib) resulted in the downregulation of EMCV internalization, indicating the involvement of both Src and Fyn in the viral entry process. However, during EMCV internalization, only Src was activated with a notable increase in its phosphorylation level, whereas Fyn and its phosphorylation level remained relatively unchanged, suggesting that Src activation plays a dominant role in this process. PP2 exhibited a downregulatory effect on both the adsorption and internalization of EMCV, which was inconsistent with the effects of Fyn overexpression and knockdown on the virus. Given that SFKs regulate diverse signaling cascades affecting viral entry-related surface proteins (12–15, 49), we hypothesize that PP2-mediated suppression of additional SFKs beyond Fyn/Src (as PP2 primarily targets LCK/Fyn) may disrupt EMCV adsorption-associated signaling. Furthermore, Fyn’s regulatory role in EMCV replication might involve alternative mechanisms, such as modulating innate antiviral responses (50).

Ezrin plays a vital role in establishing functional connections between membrane proteins and the cytoskeleton, thereby regulating membrane protein dynamics and cytoskeletal rearrangements (19). Recent studies have shown that other viruses manipulate Ezrin-related pathways to facilitate their entry into cells (15, 32, 33). Similarly, in our previous research, virus overlay protein blot assays were conducted to screen for cellular proteins that interact with EMCV viral particles. Fourteen specific proteins were obtained, including three members of the ERM family: Ezrin, Moesin, and Radixin (Y. M. Yang et al., unpublished data). In this study, cells were treated with the Ezrin inhibitor NSC668394, resulting in significant and dose-dependent inhibition of Ezrin phosphorylation. Irrespective of whether the cells were inoculated with a low or high dose of EMCV, infection was markedly inhibited under NSC668394 treatment conditions. Consistent with previous findings, the level of Ezrin phosphorylation also affects EMCV entry into cells. Furthermore, our study is the first to observe a significant enhancement in the sequential activation of Src/Ezrin/Cav-1 during EMCV infection, a phenomenon that also occurs during JEV entry (15).

The canonical cellular receptor mediating pan-species EMCV infection remains unidentified. While recent studies by Bazzone et al. (51) and Baggen et al. (52) identified ADAM9 as a critical host factor facilitating early-stage viral entry or cytoplasmic genome release in murine and human cells; its dispensability in cardiac tissue infection and post-entry replication suggests tissue-specific functionality. In a separate study, systematic antibody blockade and infection profiling across diverse cell lines established ICAM-1 as EMCV’s functional receptor for cross-species invasion (Z. Y. Hou et al., unpublished data). We further mapped critical binding interfaces to residues 39–47 (VP1 binding) and 75–83 (VP2 binding) within the N-terminal domain of ICAM-1, which facilitates viral capsid rearrangement and host cell entry. These two regions are highly conserved across species, including humans, pigs, and mice (Z. Y. Hou et al., unpublished data). Here, we found that ICAM-1 also interacts with Src. Notably, in BHK-21 cell lines with ICAM-1 knocked out, the expression of pSrc activated by EMCV was markedly diminished, resulting in subsequent decreased levels of both pEzrin and pCav-1, conditions that are unfavorable for viral internalization. These findings indicate that upon binding to the cell, EMCV relies on ICAM-1 to activate the Src-Ezrin-Cav-1 signaling pathway, thereby facilitating the viral internalization process. However, the underlying mechanism by which EMCV infection elevates Src phosphorylation levels remains elusive. Our subsequent step will involve screening and comparing the protein interactome of Src before and after EMCV infection, with the aim of pinpointing crucial proteins that bridge ICAM-1 and Src, thereby providing deeper insights into the molecular mechanisms governing EMCV internalization.

In conclusion, we have made a novel identification of the involvement of Src, Ezrin, and Cav-1 in the internalization of EMCV in BHK-21 cells. Furthermore, we have introduced ICAM-1 as a potential receptor upstream of the Src-Ezrin-Cav-1 cascade reaction. Ultimately, we have confirmed the existence of the ICAM-1-Src-Ezrin-Cav-1 signaling pathway during the entry of EMCV into BHK-21 cells. Overall, our study has primarily unveiled the molecular mechanism underlying EMCV invasion into BHK-21 cells, offering several promising new targets for the development of therapeutic interventions against EMCV.

## Data Availability

The data sets used and/or analyzed during the current study are available from the corresponding author upon reasonable request.
